# 4-Methyl-*N*-(4-methyl­phen­yl)benzene­sulfonamide

**DOI:** 10.1107/S1600536810008329

**Published:** 2010-03-10

**Authors:** Islam Ullah Khan, Shahzad Sharif, Mehmet Akkurt, Arif Sajjad, Jamil Ahmad

**Affiliations:** a Materials Chemistry Laboratory, Department of Chemistry, Government College University, Lahore 54000, Pakistan; b Department of Physics, Faculty of Arts and Sciences, Erciyes University, 38039 Kayseri, Turkey

## Abstract

In the title compound, C_14_H_15_NO_2_S, the two aromatic rings enclose a dihedral angle of 70.53 (10)°. A weak intra­molecular C—H⋯O hydrogen bond generates an *S*(6) ring motif. The crystal structure features inversion-related dimers linked by pairs of N—H⋯O hydrogen bonds.

## Related literature

For the synthesis, see: Deng & Mani (2006 ▶). For the biological activity of sulfonamides, see: Pandya *et al.* (2003 ▶); Supuran & Scozzafava (2000 ▶). For the effects of substituents on the crystal structures of and bond lengths in aryl sulfonamides, see: Sharif *et al.* (2010 ▶); Gowda *et al.* (2008 ▶, 2009 ▶, 2010 ▶); Nirmala *et al.* (2009*a* ▶,*b* ▶)·For graph-set notation, see: Bernstein *et al.* (1995 ▶); Etter (1990 ▶).
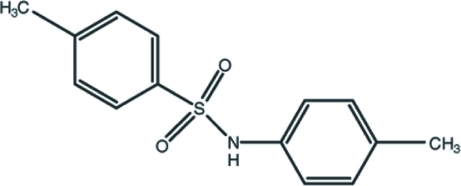


## Experimental

### 

#### Crystal data

C_14_H_15_NO_2_S
*M* *_r_* = 261.34
Triclinic, 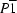

*a* = 8.6419 (8) Å
*b* = 8.8016 (8) Å
*c* = 9.2509 (7) Å
α = 88.187 (4)°
β = 77.010 (4)°
γ = 74.812 (4)°
*V* = 661.41 (10) Å^3^
*Z* = 2
Mo *K*α radiation
μ = 0.24 mm^−1^
*T* = 296 K
0.28 × 0.17 × 0.08 mm

#### Data collection

Bruker APEXII CCD diffractometer
11831 measured reflections
3259 independent reflections
2323 reflections with *I* > 2σ(*I*)
*R*_int_ = 0.038

#### Refinement

*R*[*F* ^2^ > 2σ(*F* ^2^)] = 0.044
*wR*(*F* ^2^) = 0.122
*S* = 1.01
3259 reflections
169 parameters
H atoms treated by a mixture of independent and constrained refinement
Δρ_max_ = 0.24 e Å^−3^
Δρ_min_ = −0.21 e Å^−3^

### 

Data collection: *APEX2* (Bruker, 2007 ▶); cell refinement: *SAINT* (Bruker, 2007 ▶); data reduction: *SAINT*; program(s) used to solve structure: *SHELXS97* (Sheldrick, 2008 ▶); program(s) used to refine structure: *SHELXL97* (Sheldrick, 2008 ▶); molecular graphics: *ORTEP-3 for Windows* (Farrugia, 1997 ▶); software used to prepare material for publication: *WinGX* (Farrugia, 1999 ▶) and *PLATON* (Spek, 2009 ▶).

## Supplementary Material

Crystal structure: contains datablocks global, I. DOI: 10.1107/S1600536810008329/bt5205sup1.cif

Structure factors: contains datablocks I. DOI: 10.1107/S1600536810008329/bt5205Isup2.hkl

Additional supplementary materials:  crystallographic information; 3D view; checkCIF report

## Figures and Tables

**Table 1:** Hydrogen-bond geometry (Å, °)

*D*—H⋯*A*	*D*—H	H⋯*A*	*D*⋯*A*	*D*—H⋯*A*
N1—H1⋯O2^i^	0.81 (3)	2.11 (3)	2.904 (2)	170 (2)
C4—H4⋯O1	0.93	2.45	3.049 (2)	122

Symmetry code: (i) 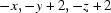.

**Fig. 1.**
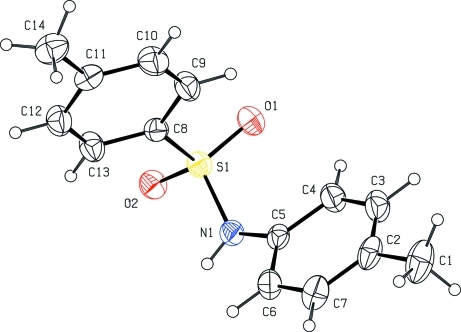
Perspective view of the title compound with the atoms labelled and displacement ellipsoids depicted at the 30% probability level for all non-H atoms.

**Fig. 2.**
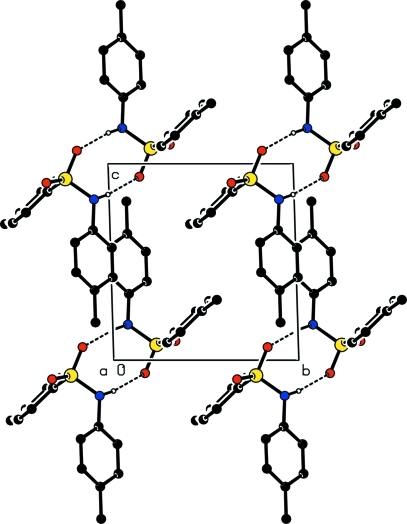
Partial packing view showing the formation of dimers through N—H···O hydrogen bonds [symmetry code: - x, 2 -y, 2 -z]. For the sake of clarity, H atoms not involved in hydrogen bonding are omitted. Hydrogen bonding is indicated by dashed lines.

## supplementary crystallographic information

### Comment

Sulfonamides are well known for their antibacterial and enzyme inhibitor properties (Pandya *et al.*, 2003). Aromatic sulfonamides were also reported to inhibit the growth of tumor cells (Supuran & Scozzafava, 2000). In continuation of our studies (Sharif *et al.*, 2010), herein, we report the crystal structure of the title compound.

The title molecule (I), (Fig. 1), is bent at the *N* atom with the C8—SO_2_—NH—C5 torsion angle of -60.71 (18)°. The dihedral angle between the two aromatic rings  is 70.53 (10)°.

The molecular conformation of the title compound is stabilized by a weak intramolecular C—H···O hydrogen bond, generating an *S*(6) ring motif (Etter, 1990; Bernstein *et al.*, 1995) (Table 1). In the crystal structure of the title compound, inversion-related molecules are linked into dimers by pairs of N—H···O hydrogen bonds, forming an *R*_2_^2^(8) graph-set motif (Table 1 and Fig. 2).

### Experimental

The synthesis of the title compound was performed by the  procedure reported by Deng & Mani (2006). 4-methyl aniline 0.535 g (5 mmol) was dissolved in 10 ml distilled water and pH of the solution was adjusted to 8 by using (3 M) Na_2_CO_3_. *p*-toluene sulfonyl chloride 0.95 g (5 mmol) was added under continuous stirring at room temperature. pH of the reaction mixture during stirring was maintained between 8-9 with 3 M Na_2_CO_3_. When the solution was clear, pH was adjusted to 2-3 using 3 M HCl. The precipitate formed was filtered and recrystallized from methanol.

### Refinement

The H atom bonded to N was refined freely. The other H atoms were positioned geometrically and were treated as riding on their parent C atoms, with C—H = 0.93-0.96 Å and *U*_iso_(H) = 1.2*U*_eq_(C) or 1.5*U*_eq_(methyl C).

### Crystal data

**Table:**

C_14_H_15_NO_2_S	*Z* = 2
*M**_r_* = 261.34	*F*(000) = 276
Triclinic, *P*1	*D*_x_ = 1.312 Mg m^−^^3^
Hall symbol: -P 1	Mo *K*α radiation, λ = 0.71073 Å
*a* = 8.6419 (8) Å	Cell parameters from 3233 reflections
*b* = 8.8016 (8) Å	θ = 2.4–25.6°
*c* = 9.2509 (7) Å	µ = 0.24 mm^−^^1^
α = 88.187 (4)°	*T* = 296 K
β = 77.010 (4)°	Rod like, light brown
γ = 74.812 (4)°	0.28 × 0.17 × 0.08 mm
*V* = 661.41 (10) Å^3^	

### Data collection

**Table:**

Bruker APEXII CCD diffractometer	2323 reflections with *I* > 2σ(*I*)
Radiation source: sealed tube	*R*_int_ = 0.038
graphite	θ_max_ = 28.4°, θ_min_ = 3.4°
φ and ω scans	*h* = −11→11
11831 measured reflections	*k* = −11→11
3259 independent reflections	*l* = −11→12

### Refinement

**Table:**

Refinement on *F*^2^	Primary atom site location: structure-invariant direct methods
Least-squares matrix: full	Secondary atom site location: difference Fourier map
*R*[*F*^2^ > 2σ(*F*^2^)] = 0.044	Hydrogen site location: inferred from neighbouring sites
*wR*(*F*^2^) = 0.122	H atoms treated by a mixture of independent and constrained refinement
*S* = 1.01	*w* = 1/[σ^2^(*F*_o_^2^) + (0.0642*P*)^2^ + 0.0376*P*] where *P* = (*F*_o_^2^ + 2*F*_c_^2^)/3
3259 reflections	(Δ/σ)_max_ < 0.001
169 parameters	Δρ_max_ = 0.24 e Å^−^^3^
0 restraints	Δρ_min_ = −0.21 e Å^−^^3^

### Special details

**Table:**

Geometry. Bond distances, angles etc. have been calculated using the rounded fractional coordinates. All su's are estimated from the variances of the (full) variance-covariance matrix. The cell esds are taken into account in the estimation of distances, angles and torsion angles
Refinement. Refinement on *F*^2^ for ALL reflections except those flagged by the user for potential systematic errors. Weighted *R*-factors wR and all goodnesses of fit *S* are based on *F*^2^, conventional *R*-factors *R* are based on *F*, with *F* set to zero for negative *F*^2^. The observed criterion of *F*^2^ > σ(*F*^2^) is used only for calculating -*R*-factor-obs etc. and is not relevant to the choice of reflections for refinement. *R*-factors based on *F*^2^ are statistically about twice as large as those based on *F*, and *R*-factors based on ALL data will be even larger.

### Fractional atomic coordinates and isotropic or equivalent isotropic displacement parameters (Å^2^)

**Table:**

	*x*	*y*	*z*	*U*_iso_*/*U*_eq_	
S1	0.18536 (5)	0.76639 (5)	0.92202 (4)	0.0448 (2)	
O1	0.34628 (15)	0.66385 (16)	0.90275 (15)	0.0579 (4)	
O2	0.10049 (15)	0.83328 (15)	1.06641 (12)	0.0537 (4)	
N1	0.1950 (2)	0.91823 (19)	0.81866 (16)	0.0471 (5)	
C1	0.4564 (3)	0.9071 (3)	0.1918 (2)	0.0860 (9)	
C2	0.3869 (3)	0.9072 (3)	0.3562 (2)	0.0589 (7)	
C3	0.4706 (3)	0.8100 (3)	0.4474 (2)	0.0635 (7)	
C4	0.4115 (2)	0.8091 (2)	0.5988 (2)	0.0570 (7)	
C5	0.2605 (2)	0.9077 (2)	0.66194 (18)	0.0423 (5)	
C6	0.1743 (2)	1.0070 (2)	0.5727 (2)	0.0559 (6)	
C7	0.2379 (3)	1.0074 (3)	0.4220 (2)	0.0661 (8)	
C8	0.0609 (2)	0.66738 (19)	0.85725 (18)	0.0428 (5)	
C9	0.1315 (2)	0.5487 (2)	0.7513 (2)	0.0608 (7)	
C10	0.0316 (3)	0.4755 (2)	0.6978 (2)	0.0668 (8)	
C11	−0.1361 (2)	0.5162 (2)	0.7502 (2)	0.0526 (6)	
C12	−0.2036 (2)	0.6345 (2)	0.8566 (2)	0.0597 (7)	
C13	−0.1067 (2)	0.7097 (2)	0.9103 (2)	0.0566 (7)	
C14	−0.2439 (3)	0.4335 (3)	0.6927 (2)	0.0702 (8)	
H1	0.118 (3)	0.994 (3)	0.843 (2)	0.068 (7)*	
H1A	0.52890	0.97510	0.17190	0.1290*	
H1B	0.36830	0.94410	0.14160	0.1290*	
H1C	0.51650	0.80190	0.15690	0.1290*	
H3	0.57180	0.74160	0.40540	0.0760*	
H4	0.47290	0.74260	0.65750	0.0680*	
H6	0.07230	1.07450	0.61420	0.0670*	
H7	0.17880	1.07680	0.36360	0.0790*	
H9	0.24500	0.51800	0.71600	0.0730*	
H10	0.07920	0.39670	0.62450	0.0800*	
H12	−0.31690	0.66430	0.89310	0.0720*	
H13	−0.15450	0.78950	0.98270	0.0680*	
H14A	−0.27990	0.48930	0.61030	0.1050*	
H14B	−0.33800	0.43100	0.77020	0.1050*	
H14C	−0.18250	0.32780	0.66110	0.1050*	

### Atomic displacement parameters (Å^2^)

**Table:**

	*U*^11^	*U*^22^	*U*^33^	*U*^12^	*U*^13^	*U*^23^
S1	0.0441 (3)	0.0466 (3)	0.0429 (2)	−0.0104 (2)	−0.0093 (2)	−0.0033 (2)
O1	0.0451 (7)	0.0608 (8)	0.0641 (8)	−0.0050 (6)	−0.0149 (6)	−0.0001 (6)
O2	0.0579 (8)	0.0586 (8)	0.0416 (6)	−0.0118 (6)	−0.0084 (5)	−0.0055 (6)
N1	0.0467 (9)	0.0452 (9)	0.0461 (8)	−0.0127 (8)	−0.0018 (6)	−0.0058 (7)
C1	0.0891 (17)	0.131 (2)	0.0475 (11)	−0.0617 (17)	0.0057 (10)	−0.0124 (12)
C2	0.0595 (13)	0.0780 (14)	0.0495 (10)	−0.0430 (11)	−0.0023 (9)	−0.0089 (10)
C3	0.0528 (12)	0.0689 (13)	0.0619 (12)	−0.0190 (10)	0.0074 (9)	−0.0175 (10)
C4	0.0472 (11)	0.0590 (12)	0.0575 (11)	−0.0080 (9)	−0.0032 (8)	−0.0046 (9)
C5	0.0404 (9)	0.0443 (9)	0.0449 (9)	−0.0197 (8)	−0.0036 (7)	−0.0069 (7)
C6	0.0432 (10)	0.0656 (12)	0.0558 (11)	−0.0151 (9)	−0.0042 (8)	0.0038 (9)
C7	0.0588 (13)	0.0898 (16)	0.0566 (11)	−0.0317 (12)	−0.0141 (9)	0.0142 (11)
C8	0.0452 (10)	0.0391 (9)	0.0426 (8)	−0.0108 (7)	−0.0070 (7)	0.0009 (7)
C9	0.0454 (11)	0.0571 (12)	0.0748 (13)	−0.0116 (9)	−0.0022 (9)	−0.0200 (10)
C10	0.0683 (14)	0.0542 (12)	0.0765 (14)	−0.0164 (11)	−0.0098 (11)	−0.0226 (10)
C11	0.0611 (12)	0.0487 (10)	0.0549 (10)	−0.0222 (9)	−0.0189 (9)	0.0085 (8)
C12	0.0452 (11)	0.0654 (13)	0.0676 (12)	−0.0160 (10)	−0.0080 (9)	−0.0067 (10)
C13	0.0465 (11)	0.0610 (12)	0.0581 (11)	−0.0120 (9)	−0.0032 (8)	−0.0160 (9)
C14	0.0783 (15)	0.0678 (14)	0.0790 (14)	−0.0320 (12)	−0.0324 (12)	0.0043 (11)

### Geometric parameters (Å, °)

**Table:**

S1—O1	1.4217 (14)	C11—C14	1.511 (3)
S1—O2	1.4324 (12)	C11—C12	1.372 (2)
S1—N1	1.6276 (16)	C12—C13	1.373 (3)
S1—C8	1.7576 (18)	C1—H1A	0.9600
N1—C5	1.429 (2)	C1—H1B	0.9600
N1—H1	0.81 (3)	C1—H1C	0.9600
C1—C2	1.504 (3)	C3—H3	0.9300
C2—C7	1.376 (4)	C4—H4	0.9300
C2—C3	1.368 (3)	C6—H6	0.9300
C3—C4	1.379 (3)	C7—H7	0.9300
C4—C5	1.376 (3)	C9—H9	0.9300
C5—C6	1.375 (2)	C10—H10	0.9300
C6—C7	1.379 (3)	C12—H12	0.9300
C8—C13	1.374 (3)	C13—H13	0.9300
C8—C9	1.373 (2)	C14—H14A	0.9600
C9—C10	1.382 (3)	C14—H14B	0.9600
C10—C11	1.374 (3)	C14—H14C	0.9600
			
S1···H4	3.0400	H1···O2^i^	2.11 (3)
O1···C4	3.049 (2)	H1A···N1^iv^	2.8000
O2···N1^i^	2.904 (2)	H1A···C5^iv^	3.0100
O2···C14^ii^	3.377 (3)	H1B···H7	2.4300
O1···H4	2.4500	H1C···H3	2.4700
O1···H9	2.6100	H3···H1C	2.4700
O1···H12^iii^	2.8900	H3···H9^vii^	2.5400
O2···H13	2.6100	H4···S1	3.0400
O2···H1^i^	2.11 (3)	H4···O1	2.4500
N1···O2^i^	2.904 (2)	H6···H1	2.3000
N1···H1A^iv^	2.8000	H6···C7^v^	3.0400
C2···C4^iv^	3.481 (3)	H7···H1B	2.4300
C4···O1	3.049 (2)	H9···O1	2.6100
C4···C2^iv^	3.481 (3)	H9···H3^vii^	2.5400
C6···C6^v^	3.595 (3)	H10···H14C	2.4400
C6···C7^v^	3.587 (3)	H12···O1^viii^	2.8900
C7···C6^v^	3.587 (3)	H12···H14B	2.4500
C14···O2^ii^	3.377 (3)	H13···O2	2.6100
C2···H14C^vi^	3.0800	H14B···H12	2.4500
C5···H1A^iv^	3.0100	H14C···H10	2.4400
C7···H6^v^	3.0400	H14C···C2^vi^	3.0800
H1···H6	2.3000		
			
O1—S1—O2	119.28 (8)	C2—C1—H1A	109.00
O1—S1—N1	108.52 (9)	C2—C1—H1B	110.00
O1—S1—C8	108.38 (8)	C2—C1—H1C	109.00
O2—S1—N1	104.26 (8)	H1A—C1—H1B	109.00
O2—S1—C8	108.41 (8)	H1A—C1—H1C	109.00
N1—S1—C8	107.41 (8)	H1B—C1—H1C	109.00
S1—N1—C5	123.93 (13)	C2—C3—H3	118.00
C5—N1—H1	112.3 (13)	C4—C3—H3	119.00
S1—N1—H1	114.3 (16)	C3—C4—H4	120.00
C1—C2—C7	121.5 (2)	C5—C4—H4	120.00
C1—C2—C3	121.5 (2)	C5—C6—H6	120.00
C3—C2—C7	117.01 (18)	C7—C6—H6	120.00
C2—C3—C4	122.9 (2)	C2—C7—H7	119.00
C3—C4—C5	119.16 (18)	C6—C7—H7	119.00
C4—C5—C6	119.03 (16)	C8—C9—H9	120.00
N1—C5—C6	118.64 (16)	C10—C9—H9	120.00
N1—C5—C4	122.18 (16)	C9—C10—H10	119.00
C5—C6—C7	120.51 (18)	C11—C10—H10	119.00
C2—C7—C6	121.4 (2)	C11—C12—H12	119.00
C9—C8—C13	119.86 (17)	C13—C12—H12	119.00
S1—C8—C9	119.76 (14)	C8—C13—H13	120.00
S1—C8—C13	120.37 (13)	C12—C13—H13	120.00
C8—C9—C10	119.12 (17)	C11—C14—H14A	109.00
C9—C10—C11	121.65 (16)	C11—C14—H14B	109.00
C10—C11—C14	121.20 (17)	C11—C14—H14C	110.00
C10—C11—C12	118.13 (17)	H14A—C14—H14B	109.00
C12—C11—C14	120.68 (17)	H14A—C14—H14C	109.00
C11—C12—C13	121.15 (17)	H14B—C14—H14C	109.00
C8—C13—C12	120.09 (16)		
			
O1—S1—N1—C5	56.26 (18)	C3—C4—C5—C6	−1.4 (3)
O2—S1—N1—C5	−175.62 (16)	C3—C4—C5—N1	−177.08 (19)
C8—S1—N1—C5	−60.71 (18)	C4—C5—C6—C7	0.3 (3)
O1—S1—C8—C13	155.34 (14)	N1—C5—C6—C7	176.04 (19)
O2—S1—C8—C13	24.51 (16)	C5—C6—C7—C2	1.3 (3)
N1—S1—C8—C13	−87.60 (15)	S1—C8—C9—C10	−177.84 (13)
O2—S1—C8—C9	−156.51 (14)	C13—C8—C9—C10	1.2 (3)
N1—S1—C8—C9	91.38 (15)	S1—C8—C13—C12	178.40 (14)
O1—S1—C8—C9	−25.69 (16)	C9—C8—C13—C12	−0.6 (3)
S1—N1—C5—C6	134.31 (16)	C8—C9—C10—C11	−1.4 (3)
S1—N1—C5—C4	−50.0 (2)	C9—C10—C11—C12	1.0 (3)
C3—C2—C7—C6	−1.5 (4)	C9—C10—C11—C14	−178.98 (17)
C1—C2—C7—C6	179.9 (2)	C10—C11—C12—C13	−0.5 (3)
C7—C2—C3—C4	0.2 (4)	C14—C11—C12—C13	179.57 (17)
C1—C2—C3—C4	178.9 (2)	C11—C12—C13—C8	0.2 (3)
C2—C3—C4—C5	1.2 (4)		

Symmetry codes: (i) −*x*, −*y*+2, −*z*+2; (ii) −*x*, −*y*+1, −*z*+2; (iii) *x*+1, *y*, *z*; (iv) −*x*+1, −*y*+2, −*z*+1; (v) −*x*, −*y*+2, −*z*+1; (vi) −*x*, −*y*+1, −*z*+1; (vii) −*x*+1, −*y*+1, −*z*+1; (viii) *x*−1, *y*, *z*.

### Hydrogen-bond geometry (Å, °)

**Table:**

*D*—H···*A*	*D*—H	H···*A*	*D*···*A*	*D*—H···*A*
N1—H1···O2^i^	0.81 (3)	2.11 (3)	2.904 (2)	170 (2)
C4—H4···O1	0.93	2.45	3.049 (2)	122

Symmetry codes: (i) −*x*, −*y*+2, −*z*+2.
